# Person-Centred Pain Measurement in the ICU: A Multicentre Clinimetric Comparison Study of Pain Behaviour Observation Scales in Critically Ill Adult Patients with Burns

**DOI:** 10.3390/ebj5020018

**Published:** 2024-06-17

**Authors:** Alette E. E. de Jong, Wim E. Tuinebreijer, Helma W. C. Hofland, Nancy E. E. Van Loey

**Affiliations:** 1 Burn Centre, Rode Kruis Ziekenhuis, Vondellaan 13, 1942 LE Beverwijk, The Netherlands; 2 Trauma Research Unit, Department of Surgery, Erasmus MC, University Medical Center Rotterdam, P.O. Box 2040, 3000 CA Rotterdam, The Netherlands; 3 Burn Centre, Maasstad Ziekenhuis, P.O. Box 9100, 3007 AC Rotterdam, The Netherlands; 4 Centre of Expertise, Urban Vitality, Faculty of Health, Amsterdam University of Applied Sciences, 1105 BD Amsterdam, The Netherlands; 5 Clinical Psychology, Faculty of Social Sciences, Utrecht University, 3584 CS Utrecht, The Netherlands

**Keywords:** pain, pain measurement, pain scale, behaviour pain scale, critical care pain observation tool, burns, intensive care, adults, clinimetrics, person-centred

## Abstract

Pain in critically ill adults with burns should be assessed using structured pain behavioural observation measures. This study tested the clinimetric qualities and usability of the behaviour pain scale (BPS) and the critical-care pain observation tool (CPOT) in this population. This prospective observational cohort study included 132 nurses who rated pain behaviour in 75 patients. The majority of nurses indicated that BPS and CPOT reflect background and procedural pain-specific features (63–72 and 87–80%, respectively). All BPS and CPOT items loaded on one latent variable (≥0.70), except for compliance ventilator and vocalisation for CPOT (0.69 and 0.64, respectively). Internal consistency also met the criterion of ≥0.70 in ventilated and non-ventilated patients for both scales, except for non-ventilated patients observed by BPS (0.67). Intraclass correlation coefficients (ICCs) of total scores were sufficient (≥0.70), but decreased when patients had facial burns. In general, the scales were fast to administer and easy to understand. Cut-off scores for BPS and CPOT were 4 and 1, respectively. In conclusion, both scales seem valid, reliable, and useful for the measurement of acute pain in ICU patients with burns, including patients with facial burns. Cut-off scores associated with BPS and CPOT for the burn population allow professionals to connect total scores to person-centred treatment protocols.

## 1. Introduction

Pain is a well-known care problem following burns. Burn pain can be long-lasting, has a fluctuating course, and is related to extensive and repetitive daily wound care procedures. A distinction is made between background pain and procedural pain. Background pain, experienced while resting, is caused immediately postburn when the inflammatory response is initiated. Procedural pain is caused by every manipulation involving the burns, which leads to additional stimulation of the nociceptors [1,2]. Adequate management of this burn pain, amongst other things, may reduce sensitivity to pain over time, the development of neuropathic pain [3], wound healing delay [4], and the development of delirium in ICU patients [5]. Adequate, person-centred, individualised pain treatment should therefore have high priority in daily burn care practice.

To evaluate the adequacy of individualised pain treatment, pain should be measured. Although valid and reliable pain measurement instruments are available for the largest groups of patients represented in the burn centres (e.g., adults able to provide self-reports, young children) [6,7,8,9,10], little research has been carried out regarding instruments for critically ill adults, often in need of mechanical ventilation and therefore unable to provide self-reports of pain. For these patients, structured behavioural observation measures are suggested, like the behaviour pain scale (BPS) and the critical care pain observation tool (CPOT) [11,12]. The Dutch versions of these scales have been validated for their use in various ICU settings [13,14,15]. Regarding BPS, as far as we know, no research in the burn setting is available. CPOT, however, has been investigated in patients with burns, although in non-ventilated patients able to self-report, and in ventilated patients with the aim to understand how the use of CPOT affected clinical judgement processes and analgesia administration [16,17]. Therefore, there is a need for more clarification regarding the clinimetric properties and clinical usability for the use of BPS and CPOT in burn care. 

One specific reason for testing the scales in patients with burns relates to the presence of facial burns that may compromise the reliability of the item facial expression. Both scales incorporate facial expression as an item to be observed, which may be hampered by facial burns, crusts, oedema, bandages, and fixation material. Another reason that requires testing in the burn setting relates to the documentation that the scales are suitable for short-time observations, usually two minutes. Procedural pain caused by wound care, however, spans a larger time frame, but multiple assessments and registrations during wound care by the nurses would be impractical. Given these burn-specific deviations from existing patient populations, more research is needed to evaluate the reliability of both scales in burn settings. In this way, person-centred, individualised pain management can be preserved. Once provided with a clinimetric sound measure, health care professionals can use this measure that enables one to tailor and evaluate pain treatment in daily burn care practice, to investigate effects of pain interventions, and thus contribute to optimal wound healing and quality of life during hospitalisation and after discharge.

The aim of this study is therefore to assess validity, reliability, clinical utility, and cut-off scores of BPS and CPOT in order to measure procedural and background pain in adult critically ill patients with burns. The research questions are as follows: (1) Are the BPS and CPOT valid, reliable, and clinically useful to measure procedural and background pain in critically ill adults with burns? (2) What are their cut-off scores that can be connected to treatment protocols?

## 2. Materials and Methods

This is a prospective observational multicentre cohort study that, although pain behaviour observation is not a patient-reported outcome, follows the consensus-based standards for the selection of health measurement instruments (COSMIN) study design checklist [18]. The study was approved by the regional medical ethics committee and the review boards of the participating hospitals and has been carried out in accordance with the Code of Ethics of the World Medical Association (Declaration of Helsinki). Patients’ relatives received written and verbal information about the study explaining that pain was measured with the usual observational pain measure and with one extra observational measure. They were assured that tailored pharmacological pain treatment remained unchanged, that the scales were subject of investigation and not the patient, and that the study would not cause any burden to the patient.

### 2.1. Participants

Patients were ≥18 years old, critically ill with burns, unable to provide self-reports of pain, mechanically ventilated, weaning from mechanical ventilation, or were recently extubated. Patients did not have developmental delays and/or limb paralysis. Participating nurses were registered burn care and/or ICU nurses employed in the burn centre of the Rode Kruis Ziekenhuis in Beverwijk, the Maasstad Hospital in Rotterdam, and the Martini Hospital in Groningen, The Netherlands. 

### 2.2. Measures

The BPS [19] and CPOT [20] are frequently investigated in various types of adult patients, both intubated and non-intubated, who are unable to self-report pain, whose motor function is intact, and whose behaviours are observable. According to international clinical practice guidelines for adult critical care patients, both scales demonstrated the best validity and reliability for monitoring pain when compared to other scales [11,12]. In this study, we used the Dutch versions as investigated by Stilma et al. [13] and Rijkenberg et al. [14].

#### 2.2.1. BPS

The BPS consists of the following 3 items: (1) facial expression; (2) upper limp movements; (3) compliance with mechanical ventilator or vocalisation. Each item has four answer categories, ranging from 1 to 4. The total score varies from 3 (no pain) to 12 (most pain). Regarding content validity, consensus on relevant pain behaviour items was reached by registered nurses [21]. Good construct and criterion validity, internal consistency, and inter-rater reliability have been reported [12,19,22,23,24,25], as well as responsiveness [26,27]. The BPS has been previously evaluated for ease of use [14].

#### 2.2.2. CPOT

The CPOT evaluates the following four behavioural items: (1) facial expression; (2) movements; (3) muscle tension; (4) compliance with ventilator or vocalisation. Each item has 3 answer categories and is scored 0, 1, or 2. The total score can range from 0 (no pain) to 8 (most pain). Content validity of the CPOT has been assessed for general ICU patients by deriving pain indicators from existing instruments, by using chart reviews of medical files, by focus groups with critical care nurses and physicians, and by questionnaires on the relevance of the yielded items [28]. Good construct and criterion validity, and internal consistency and inter-rater reliability have been reported [12,14,20,24,29,30,31,32,33,34,35]. Furthermore, fair criterion validity has been described in a meta-analysis [33]. The CPOT was also investigated in patients with burns who were alert and able to self-report. Good criterion validity, high internal consistency, but poor inter-rater reliability were reported in the burns population [34]. 

#### 2.2.3. Clinical Utility Questionnaire

To survey the clinical usefulness of the scales from nurses’ points of view, we used the clinical utility questionnaire, a structured closed-ended self-report questionnaire using a 5-point Likert scale [36]. It includes items about the extent of the scales in providing clinically useful patient information and readily understandable scores. In addition, items about ease of use, time required and clarity of the scales, and the property of the scales to adapt the total score to pain treatment were included. The degree of the severity of pain, the ability to differentiate between no pain and unbearable pain, and the relevance of the scale items were questioned as well. The questionnaire also includes questions about the relevance of the scale items (reflection of pain-specific features). The results of these questions are used to assess content validity. The questionnaire was completed by nurses that participated in the observations at the end of the study.

### 2.3. Data Collection Procedure

Before this study, nurses were already familiar with the use of BPS or CPOT. Using a standardised one-hour educational programme about pain and pain assessment, the study procedure, and the use of scales, nurses were trained before taking part in the study. Instruction cards were available in the patient rooms. Patients who met the inclusion criteria (consecutive sample) were simultaneously observed by means of the BPS and CPOT three times a day by two nurses who kept independent records. The pairs of assessing nurses were assigned by convenience. Due to shifts, variation in the composition of the pairs that were assigned to different patients per day, and thus variation in observers, was considerable. Background pain was recorded in the morning, at least one hour before wound care, and in the afternoon, at least one hour after wound care (in accordance with usual daily practice). Patients were observed during two minutes. Procedural pain was assessed directly after wound care. Nurses were asked to rate the overall procedural pain of the entire wound care procedure. These measurement points belong to the standard procedure, as convened between the burn centres. The data collection forms were integrated in the electronic patient file. The scales were ordered differently for each data collection point and for the individual nurse to vary the order of completion of the scales. Nurses were requested not to discuss or compare their individual ratings.

Furthermore, we retrieved the following information from the patient files: age, gender, and extent and cause of the burns. The number of surgical procedures and length of stay in the ICU were obtained at the conclusion of the patients’ hospitalisation.

Characteristics of the participating nurses we recorded were age, gender, education, and number of years working in burn care. Patients and nurses were encoded. 

### 2.4. Data Analysis

Classical test theory according to the COSMIN guideline [18] was applied to assess the clinimetric properties on item level. Validity was determined by content validity (relevance of the scale items as reported by nurses), construct validity (all items are manifestations of the same underlying construct), and criterion validity (how scales correlate with other measures of the same construct). These types of validity were assessed by descriptive statistics, principal component factor analysis (PCA), confirmatory factor analysis (CFA), and Spearman’s rho, respectively. 

Reliability was assessed by internal consistency (the degree in which the items of the scale belong to the same concept) and inter-rater reliability (the degree in which observers assign the same ratings). The internal consistency was calculated by Cronbach’s alpha. Inter-rater reliability was calculated by intraclass correlation coefficients (ICC). The model chosen for ICC was two-way random, absolute agreement, single measures, 95% confidence interval, test value 0. 

To assess cut-off scores, we performed receiver operating characteristic analysis (ROC). We used descriptive statistics for all other analyses.

Data were analysed with the statistical program SPSS 25.0 (SPSS Inc., Chicago, IL, USA), except for principal component and confirmatory factor analysis (Mplus version 6.1, Muthén and Muthén) and ROC curves (MedCalc^®^ Statistical Software version 20.100, MedCalc Software Ltd., Ostend, Belgium; 2022). Three researchers (A.D.J., W.T., and N.V.L.) were involved in the data analyses. We used the COSMIN criteria for good measurement properties to evaluate our results [37].

## 3. Results

Data were collected from July 2018 to January 2020. The number of patients included in the study was 75. In agreement with clinical practice guidelines [12], in general, fentanyl, sufentanyl, remifentanil, midazolam, and/or propofol were used for pain and sedation control in patients on the ventilator, with increased dosages during wound care. Acetaminophen, naproxen, and oxycodon (slow release for background pain and fast release for procedural pain) were used for pain control in patients that were on the ventilator. Included patients were observed by a total of 132 nurses, assigned by convenience, depending on the team composition per shift. COSMIN suggests that a good sample size implies 50–99 patients and at least 30–49 professionals [37]. We collected 2210 observations with BPS, of which 1040 were paired observations, and 2322 observations using CPOT, of which 1128 were paired. The extent of pain behaviour is, per scale, and per type of pain, presented in Table 1. Although means and medians were low, implying that pain may be well managed, all answer categories were used, except for ‘unable to control ventilation’ in BPS. 

### 3.1. Characteristics

Regarding the patients, 75% were male, mean age was 50 years (SD 18). Causes of the burns were flame (38%), hot liquids (44%), and others, e.g., electric, chemical (18%). The mean total burned body surface area was 27% (SD 20), and 38% of the patients had a facial burn. The mean number of days on the ICU was 22 (SD 18), and the mean number of days on the ventilator was 15 (SD 15). Regarding the nurses, 75% were female, and their mean age was 44 years (SD 12). Amongst them, 23% had <1 year experience in burn care, 20% 1–5 years, and 57% >6 years of experience. Variation in the composition of the pairs of observing nurses was, due to shifts, substantial.

### 3.2. Content Validity 

The majority of nurses agreed that the scale items reflect background and procedural pain-specific elements appropriately and that all items are relevant. Of the nurses, 63% agreed that the BPS reflects background pain, and 72% agreed that the BPS reflects procedural pain. Regarding the CPOT, 87% indicated that this scale reflects background pain features, and 80% agreed that procedural pain features were well reflected. These findings also imply that CPOT showed more agreement than BPS items. Nurses did not provide suggestions for adding or deleting items and considered the scales as complete. 

### 3.3. Construct Validity

For BPS, PCA in ventilated patients showed that all items loaded on one factor, accounting for 63% of the variance. Factor loadings for the three items, i.e., facial expression, movement, and compliance with the ventilator, were 0.82, 0.83, and 0.72, respectively (Table 2). CFA testing a single factor produced a well-fitting model with RMSEA = 0, CFI = 1, and TLI = 1. PCA in non-ventilated patients yielded one factor accounting for 68% of the variance. Factor loadings for facial expression, movement, and vocalisation were 0.83, 0.85, and 0.79, respectively. CFA was not possible to calculate due to a correlation of 1.0 between the items facial expression and movement.

For CPOT, PCA in ventilated patients showed that all items loaded on one factor, accounting for 62% of the variance. Factor loadings for the four items, i.e., facial expression, movement, muscles, and compliance with the ventilator, were 0.83, 0.81, 0.82, and 0.69, respectively. CFA was performed using a WLSMV estimator. The model testing a single factor model produced adequate fit indices: RMSEA = 0.043 (90% confidence interval 0.000–0.088), CFI = 0.998, TLI = 0.995, with estimates ranging from 0.96 to 0.80. PCA in non-ventilated patients showed that all items loaded on one factor, accounting for 56% of the variance. Factor loadings of the items facial expression, movement, muscles, and vocalisation were 0.81, 0.73, 0.80, and 0.64, respectively. CFA was performed using a WLSMV estimator. The model testing a single factor model partly produced adequate fit indices with CFI = 0.989 and TLI = 0.967, but RMSEA = 0.103 (90% confidence interval 0.036–0.182) indicated moderate model fit as RMSEA preferably is <0.08 but the model has acceptable CI values. Estimates ranged from 0.86 to 0.67. 

Both scales show that the items are strongly associated. Furthermore, all BPS and CPOT items loaded on one latent factor, suggesting that the items measure the same construct and can be used in the burn setting. 

### 3.4. Criterion Validity

Spearman’s rho correlation coefficients between BPS and CPOT, both in ventilated and non-ventilated patients, are shown in Table 3. As the BPS and CPOT correlate for both types of pain (Spearman’s rho ≥ 0.70) [37], they probably measure the same construct.

### 3.5. Internal Consistency 

Internal consistency results of the BPS and CPOT are presented in Table 4. The coherence between the items was good (Cronbach’s alpha ≥ 0.70) [37], except for non-ventilated patients observed by BPS, which was just below 0.70. An item contributes to a scale if alpha, when calculated after this item is deleted, has a lower value than alpha of the entire scale. All alpha values were lower when items were deleted, suggesting that all behaviour items belong to the scales. The CPOT showed higher alphas than the BPS, proposing that the CPOT items show more coherence.

### 3.6. Inter-Rater Reliability

To test if two nurses observe similar pain behaviour in one patient at the same time, we calculated ICCs of the total scores. As presented in Table 5, ICCs for the BPS and CPOT met the criterion of ≥0.70 [37], with small confidence intervals, but were slightly insufficient in patients with facial burns, i.e., 0.63 end 0.66 for BPS and CPOT, respectively. This suggests that facial burns, bandages, oedema, and/or crusts may complicate the observation of facial expression for both scales to some extent.

### 3.7. Clinical Usefulness

Of the participating nurses, 41% (N = 54) responded to the clinical usability questionnaire. The majority of the nurses indicated that both the BPS and the CPOT provide clinically useful information (82% and 74% of the nurses for BPS and CPOT, respectively), they are easy and quick to complete (87% and 76%), clear and easy to understand (85% for both scales), and that total scores can be connected to individualised pain treatment (63% and 57%). 

### 3.8. Cut-Off Scores

BPS score 4 and CPOT score 1 have the highest probability to detect all patients suffering ‘unacceptable pain’, see Table 6. At these points, the probability of undertreated pain is lowest, suggesting that patients with higher scores may need pain treatment evaluation.

## 4. Discussion

This is the first study that investigated the validity (content, construct, and criterion), reliability (internal consistency, inter-rater reliability), and clinical usefulness of two structured pain behavioural observation scales in critically ill adults with burns. We also calculated cut-off scores for both scales.

BPS total scores can range from 3 to 12, while CPOT total scores range from 0 to 8. The mean pain scores were low for background pain (BPS 3.7, CPOT 1.0), as well as for procedural pain (BPS 3.9, CPOT 1.2). The low total scores and the small difference between the two types of pain may be due to intravenous sedation during wound care. Furthermore, during wound care, nurses are allowed to administer additional intravenous pain medication and can thus directly anticipate observed pain behaviour. This study suggests therefore that pain may be well managed in our patients with burns in the ICU. Mean low pain scores in various types of ICU settings for background pain by BPS (total scores of 3 and 4) and for procedural pain (total scores of 5, 6, and 7 for application of compression stockings, tracheal suction, venipuncture, turning, positioning, or eye cleaning) have been reported [14,15,19,22,24,26,27]. Compared to wound care, these are all procedures of short duration, probably without application of extra pain or sedation medication. Also, low CPOT scores for background pain (total scores of 0, 1, and 2) and procedural (total scores of 1, 2, 3, and 4 for positioning, turning, mediastinal tube removal, and tracheal suction, respectively) are described [14,15,20,24,29,33,34,35]. In the burn setting, background pain total score 0 and procedural pain score 1 (wound care and occupational therapy) obtained by CPOT have been reported [16] and are similar to our results.

Regarding content validity, the majority of nurses believe that the items of both scales reflect background and procedural pain-specific elements appropriately and that all items are relevant. This corresponds with a recent update of the clinimetric properties of behavioural pain assessment tools for noncommunicative critically ill patients [38]. No changes in items of BPS and CPOT have been proposed.

All behaviour items were related to each other and refer to one construct, for ventilated patients but also for non-ventilated patients. For BPS, similar results have been reported earlier, i.e., explained variance of 55%, factor loadings of 0.79 for facial expression, 0.79 for movement, and 0.63 for compliance with the ventilator [19], and explained variance of 65%, factor loadings of 0.90 for facial expression, 0.85 for movements, and 0.64 for compliance with the ventilator [22]. However, these studies used compliance with the ventilator item only. The vocalisation item was not investigated. Regarding CPOT, no principal component factor analyses have been described in previous research. 

Regarding the correlation coefficients between the BPS and CPOT, positive and high correlations for both types of pain were also reported by other researchers comparing these two scales (r > 0.80, *p* < 0.05) [39,40]. 

In previous research, internal consistency varied, with ranges from 0.60–0.83 for BPS and 0.31–0.81 for CPOT [25]. A psychometric analysis update by Gelinas et al. states that internal consistency of BPS and CPOT was good (Cronbach’s alpha ≥ 0.70) in most of the 19 studies that had been reviewed [38]. In the burn population, an alpha of 0.67 was found for CPOT [16]. In our study, the coherence between the items of the BPS and CPOT was good but was just below 0.70 for non-ventilated patients observed by BPS. As the vocalisation item showed lower factor loadings, this may indicate that particularly this item deviates from other items when observing pain behaviour. It should be noted that most of the previous research included ventilated patients and did not use the version with the vocalisation item. Furthermore, the CPOT showed higher alphas than the BPS, proposing that the CPOT items show more coherence. Cronbach’s alpha may be affected by the number of items and can be higher when a questionnaire is composed of more items [6]. However, in other studies comparing those two scales, alphas of BPS and CPOT were similar (BPS 0.80, CPOT 0.81 [24], BPS 0.70, CPOT 0.71 [15], BPS 0.62, CPOT 0.62 [14]).

ICCs for the BPS and CPOT met the criterion of ≥0.70 but were slightly insufficient in patients with facial burns. A distinction between patients with facial and no facial burns has not been made in earlier CPOT research in the burn population, but we assume that facial burns, oedema, crusts, and bandages may have influenced inter-rater agreement to some extent. Also, training of nurses may have influenced the high ICCs. When compared to other research, moderate to high ICCs were found in various ICU settings (BPS 0.74–0.94, CPOT 0.72–0.93 [25], BPS 0.80–0.97, CPOT 0.52–0.88 [41], BPS > 0.60, CPOT > 0.60 [38]. Furthermore, inter-rater reliability for CPOT in the burn population was found to be poor (0.43 k coefficient), with low CPOT total scores between 0 and 1 [16], while it may be expected that it is easier to agree when pain behaviour scores are low. 

Nurses agreed on the clinical usefulness of the scales. Evaluating the BPS, the majority of professionals (86%) were satisfied with its ease of use [19]. In an overview of Varndell et al. [25], 73% of the respondents considered CPOT helpful for daily practice, 100% rated CPOT clear and simple, and 79% quick to use. In our study, BPS showed slightly higher agreement percentages than CPOT, but it should be noted that 83% of the nurses who completed the clinical usefulness questionnaire used the BPS in daily practice, indicating that this scale was familiar. A preference for BPS above CPOT was also reported by Chanques et al. [24]. 

We found low cut-off scores for both scales, namely 4 for BPS and 1 for CPOT, that may be explained by adequate sedation and analgesia. Similar low cut-off scores have been reported. In an overview of the literature, cut-offs of 4 and 5 for BPS (3–12 scale) were reported, and 1, 2, and 3 for CPOT (0–8 scale) [14]. In another overview, CPOT cut-offs between 2 and 3 have been found [25,42], with sensitivity between 67% and 86% and specificity between 78% and 83% [25]. In research led by Gelinas [38], BPS cut-offs are between 5 and 7 (sensitivity between 52% and 90%, specificity between 46 and 92%). For the CPOT, cut-offs between 1 and 2 (sensitivity 47–81%, specificity 65–97% for background pain are reported, and for procedural pain between 2 and 3, sensitivity 67–93%, specificity 46–90%).

Finally, a limitation of this study may relate to the lack of delirium and agitation measurements, which could have been confounding factors. We have not taken measurements from CAM-ICU or RASS into account in this study since these are other constructs with different items to observe. 

## 5. Conclusions

We investigated two pain behaviour observation instruments, the BPS and the CPOT, for use in the measurement of acute pain in critically ill patients with burns. Background and procedural pain can be assessed with the currently available measurement instruments; the CPOT and BPS showed good validity and reliability in this study and are considered clinically useful. Although ICCs for patients with facial burns were just below the COSMIN criterion, this difference was only minimal, so we assume that both the BPS and the CPOT can be used in people with a facial burn. Furthermore, although the CPOT has better clinimetric properties, both measures seem to meet the COSMIN criteria and to be suitable for use in the burn ICU, also according to the nurses. This implies that pain management can now be reliably evaluated in critically ill patients with burns. We recommend the use of these existing scales that were originally developed for general ICUs instead of developing new scales. In the context of uniformity and future research into the effects of pain-relieving interventions, it is recommended to use the same instrument for each burn centre. The development of pain management protocols is recommended in order to connect them to the total scores of the scales. We suggest further research of the clinimetric properties of the scales in patients with high delirium and/or agitation scores, since high scores may lead to additional use of analgesics where anti-delirium interventions would be more appropriate.

## Figures and Tables

**Table 1:** Extent of pain behaviour.

Type of Pain	BPS	CPOT
Background		
Mean (SD)	3.7 (1.2)	1.0 (1.5)
Median (IQR)	3 (3–4)	0 (0–1)
Min–Max	3–11	0–8
Procedural		
Mean (SD)	3.9 (1.3)	1.2 (1.7)
Median (IQR)	3 (3–5)	1 (0–2)
Min–Max	3–11	0–8

BPS: behaviour pain scale, total score ranges from 3 (no pain) to 12 (most pain); CPOT: critical care pain observation tool, total score ranges from 0 (no pain) to 8 (most pain); IQR: from 25th to 75th interquartile ranges.

**Table 2:** Principal component factor analysis.

	BPS V	BPS N	CPOT V	CPOT N
Explained variance	63%	68%	62%	56%
Factor	Factor loadings			
Facial expression	0.82	0.83	0.83	0.81
Movements	0.83	0.85	0.81	0.73
Muscle tension	NA	NA	0.82	0.80
Compliance ventilator	0.72	NA	0.69	NA
Vocalisation	NA	0.79	NA	0.64

BPS: behaviour pain scale; CPOT: critical care pain observation tool; V: ventilated; N: non-ventilated; NA: not applicable (muscle tension is not a BPS item; no vocalisation when on ventilator).

**Table 3:** Correlation coefficients BPS-CPOT.

	Spearman’s Rho	N Observations	*p*
BP V	0.81	1124	0.00
BP N	0.73	270	0.00
PP V	0.85	637	0.00
PP N	0.86	151	0.00

BPS: behaviour pain scale; CPOT: critical care pain observation tool; V: ventilated; N: non-ventilated; BP: background pain; PP: procedural pain.

**Table 4:** Internal consistency.

Cronbach’s α			
		BPS	CPOT
Background pain	V	0.71	0.79
	N	0.67	0.79
Procedural pain	V	0.71	0.83
	N	0.67	0.87

BPS: behaviour pain scale; CPOT: critical care pain observation tool; V: ventilated; N: non-ventilated.

**Table 5:** Inter-rater reliability.

Intraclass Correlation Coefficient Total Scores (95% Confidence Interval)					
BPS			CPOT		
Overall	Without Facial Burns	With Facial Burns	Overall	Without Facial Burns	With Facial Burns
**0.70** (0.66–0.73)	**0.72** (0.68–0.76)	0.63 (0.57–0.69)	**0.71** (0.68–0.73)	**0.73** (0.69–0.76)	0.66 (0.60–0.71)

BPS: behaviour pain scale; CPOT: critical care pain observation tool.

**Table 6:** Cut-off scores.

	Criterion Values of ROC Curve				
Scale	Cut-Off Score	Sensitivity (%)	95% CI	Specificity (%)	95% CI
BPS	4	54	48–60	65	62–68
CPOT	1	55	51–58	73	69–77

ROC: receiver operating characteristic analysis; Sensitivity: the ability of a scale to correctly identify patients with pain; CI: confidence interval; Specificity: the ability of a scale to correctly identify people without pain; BPS: behaviour pain scale; CPOT: critical care pain observation tool.

## Data Availability

The participants of this study did not give written informed consent for their data to be shared publicly, so data are not available.
